# A One Health Platform for Future Epidemic Preparedness

**DOI:** 10.3390/idr16020023

**Published:** 2024-03-20

**Authors:** Francesco Branda, Fabio Scarpa, Nicola Petrosillo, Massimo Ciccozzi

**Affiliations:** 1 Unit of Medical Statistics and Molecular Epidemiology, Università Campus Bio-Medico di Roma, 00128 Rome, Italy; m.ciccozzi@unicampus.it; 2 Department of Biomedical Sciences, University of Sassari, Viale San Pietro 43b, 07100 Sassari, Italy; fscarpa@uniss.it; 3 Infection Prevention & Control/Infectious Disease Service, Fondazione Policlinico Universitario Campus Bio-Medico, 00127 Rome, Italy

**Keywords:** open data, public health, virus surveillance, epidemiology, one health

## Abstract

Here, we introduce the EpiConnect Intelligence Platform (ECIP), a platform facilitating rapid, transparent data sharing and analysis to support researchers and public health officials in Europe, with a focus on Italy. ECIP provides reliable, concise, machine-readable data to aid in epidemiological understanding, standardize case characteristics, and estimate key parameters. The platform adheres to FAIR (findable, accessible, interoperable, reusable) principles, offering easily accessible and downloadable datasets for researchers’ endeavors. Future enhancements include involving national public health authorities, expanding data streams, and fostering collaboration between experts and users for improved epidemic risk monitoring. Shared standards among diverse surveillance systems are advocated to achieve common strategic goals, emphasizing the need for forward-looking policies to empower professionals to analyze disease dynamics in the context of evolving health crises. The recent emergencies underscore the importance of collective efforts towards shared strategic goals, highlighting the necessity for coordinated action to address mutual concerns affecting everyone’s lives.

## 1. Introduction

Threats related to pandemics and epidemics are recurring phenomena that are largely underestimated, and actions to prepare for future threats suffer severe underinvestment. The emergence of SARS-CoV-2 and the subsequent development of the COVID-19 pandemic have highlighted the complexities in comprehending the dynamics of risk during an emerging infectious disease event. Once the confirmation of a public health incident is established, it becomes imperative to conduct risk assessments, enhance surveillance capabilities, and gather intelligence to gain insights into the unfolding situation. This process helps evaluate the effectiveness of control measures and formulate strategies to address various potential scenarios that may arise. Traditional surveillance methods, such as monitoring case and mortality numbers, prove insufficient when dealing with such multifaceted public health challenges.

The emergence of COVID-19 posed an unprecedented challenge to the medical community and policymakers due to the lack of sufficient data and evidence to formulate effective strategies against the virus. The urgency of the situation underscored the need for timely communication of crucial information, facilitating access to and analysis of large datasets. An important avenue for gaining valuable information has been the analysis of electronic health records (EHRs), which capture patient data in real-time from routine clinical care. This approach has proven critical not only for improving disease surveillance but also for monitoring the effectiveness of emerging treatments. The ability to draw insights from real patient experiences has been particularly useful in adapting strategies to the evolving nature of the pandemic [1]. In addition, the rapid development and dissemination of COVID-19 vaccines marked a watershed moment in the global response to the virus [2]. These vaccines, licensed for use in emergencies on the basis of limited clinical data, owe much of their success to the critical role played by real-world data, which are critical to understanding vaccine efficacy and safety in diverse populations [3]. Efforts to address these challenges and establish frameworks for responsible data sharing are crucial. Encouraging collaboration and establishing standardized protocols for handling and sharing real-world data could pave the way for a more coordinated and effective response to future public health crises. As the world continues to navigate the complexities of the COVID-19 pandemic, leveraging the potential of real-world data remains a key strategy for informed decision-making and mitigating the impact of emerging health threats.

In the increasingly complex landscape of epidemiological surveillance and the management of emerging infectious diseases, several health platforms have played a key role in providing tools and resources to monitor, analyze, and respond to these global threats. Among them, Nextstrain (https://nextstrain.org/, accessed on 28 February 2024) stands out as a leading platform for monitoring the genomic evolution of pathogens, offering interactive visualizations and analysis tools to understand patterns of disease spread. HealthMap (https://www.healthmap.org/en/, accessed on 28 February 2024), founded in 2006, has revolutionized the way infectious diseases are detected by using data mining algorithms to analyze online public information sources. GIDEON (https://www.gideononline.com/, accessed on 28 February 2024), a landmark in infectious disease surveillance since 1992, provides detailed and up-to-date information on infectious diseases, including epidemiological data and treatment guidelines. Similarly, ProMED-mail (https://promedmail.org/, accessed on 28 February 2024), founded in 1994, is a global network of infectious disease experts that monitors and reports on outbreaks and emerging infectious diseases worldwide. Finally, FluTrackers (https://flutrackers.com/forum/, accessed on 28 February 2024), launched in 2006, is an online community of infectious disease and influenza experts that provides real-time updates and in-depth analysis on influenza outbreaks and other emerging infectious diseases.

## 2. Proposed Platform

**Figure 1:**
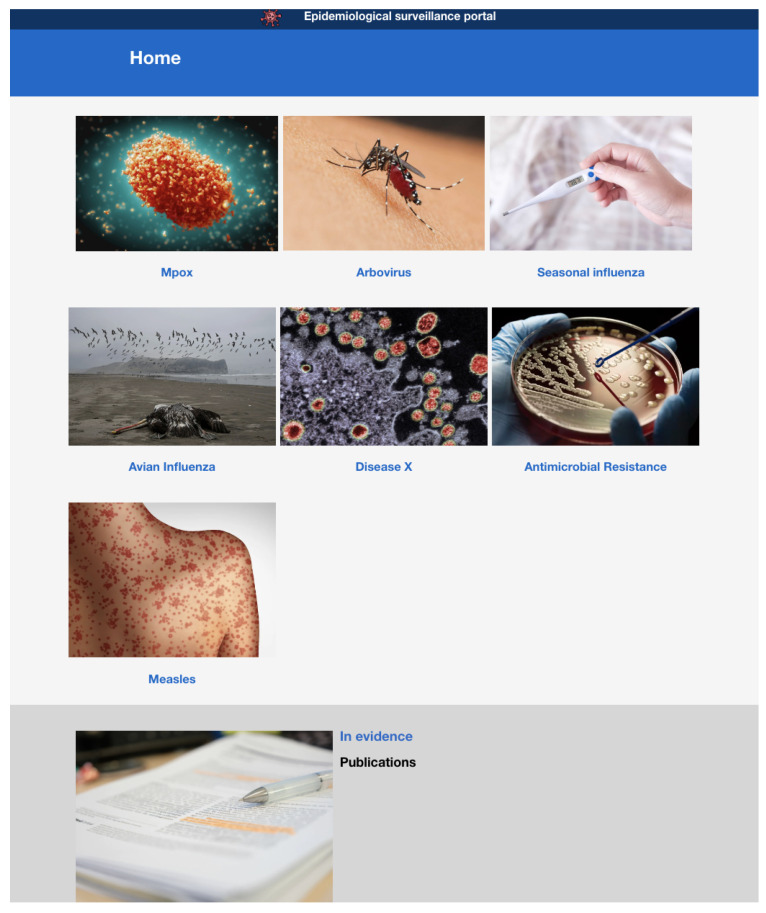
ECIP webpage.

This paper introduces the EpiConnect Intelligence Platform (ECIP) (Figure 1), a web-based platform available at the link: https://tinyurl.com/episorveglianza (accessed on 28 February 2024) that enables health professionals and scientists to contextualize, analyze, and verify information [4,5,6] for rapid, evidence-based action on real data. Created in 2022, through the collaborative effort of virologists, epidemiologists, statisticians, data scientists, and molecular biologists, ECIP aims to achieve three key objectives: (i) to support epidemiological understanding by providing key data to understand the origins and transmission dynamics of a disease to improve the ability to anticipate and respond to epidemiological emergencies [7,8,9]; (ii) to harmonize information across countries, standardize it according to strict principles and share it in an accessible manner [10] to create a uniform and reliable information base; (iii) to estimate key epidemiological parameters, such as incubation period, serial interval, and reproductive number, providing crucial information for understanding and managing infectious diseases [11].

The success of the platform is evidenced by the publications produced and available at http://tinyurl.com/episorveglianza-publications (accessed on 28 February 2024) and measurable by the numerous citations received by the published papers. However, although ECIP shares some features with the other platforms mentioned above, it also has unique advantages that distinguish its approach. For example, ECIP integrates data from a wide range of sources, including reports from governments and public health organizations, news media reporting of health official statements, and real-time surveillance data, providing a more comprehensive view of the epidemiological situation. ECIP is designed with a strong emphasis on interoperability and collaboration, facilitating the exchange and sharing of data among actors at the local, national, and international levels. This collaborative framework allows ECIP to leverage the collective expertise and resources of different actors, leading to more informed decision-making and coordinated responses to public health threats. In addition, ECIP prioritizes accessibility and ease of use for users, offering a friendly interface that allows health professionals and scientists to contextualize, analyze, and verify information quickly. Through its intuitive design, ECIP aims to democratize epidemic intelligence and provide users with the tools they need to act based on real-time evidence.

**Figure 2:**
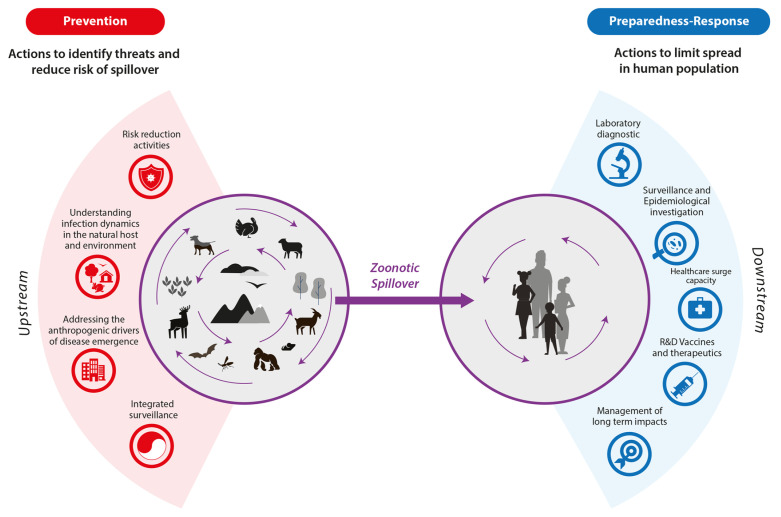
Framework for pandemic prevention, preparedness, and response [12].

Figure 2 describes the conceptual framework on which the ECIP platform for pandemic prevention, preparedness, and response is based. This approach aims to shift the infectious disease control paradigm from reactive to proactive, focusing on primary prevention. Primary prevention involves addressing the causes of disease outbreaks, which include ecological, meteorological, and anthropogenic factors and activities that increase the risk of spread, ultimately reducing the likelihood of human infection. Key elements of the primary prevention strategy include:

1. Understanding infection dynamics in the natural host and environment: Conduct comprehensive studies to unravel the complexities of pathogen interactions within natural hosts and ecosystems. This involves studying modes of transmission, reservoir species, and environmental factors that influence pathogen persistence and evolution.
2. Addressing the anthropogenic drivers of disease emergence: Identify and mitigate human-induced factors that contribute to disease outbreaks. This includes analyzing activities such as deforestation, urbanization, agricultural practices, and wildlife trade that can alter ecological balances and facilitate the spread of pathogens from animals to humans.
3. Risk reduction activities: Implement targeted measures to reduce the risk of disease transmission. This includes public health campaigns to educate communities, sustainable agricultural practices, enhanced biosecurity measures in high-risk areas, and promotion of responsible interactions between humans, animals, and the environment.
4. Integrated surveillance: Take a holistic approach to data collection, integrating information from different sources. This includes monitoring human and animal health, environmental conditions, and socioeconomic factors. Use advanced technologies such as real-time data analysis and artificial intelligence for effective surveillance to detect potential disease outbreaks early and support a coordinated response.

Preparedness–response is a critical phase in public health management, focusing on proactive measures to anticipate, prepare for, and respond to potential health emergencies, including infectious disease outbreaks. This phase is characterized by strategic planning, resource allocation, capacity building, and rapid, coordinated actions when faced with an emerging health threat. Key elements integral to this step include:

1. Laboratory diagnostics: A crucial element of this phase is the implementation of advanced and timely laboratory diagnostic systems. The ability to rapidly identify the pathogens responsible for an outbreak is critical to an effective response. This involves the rapid development and distribution of reliable diagnostic tests.
2. Surveillance and epidemiological investigation: An effective response requires active surveillance and thorough epidemiological investigation. Monitoring and understanding the spread of the pathogen is essential to identify outbreaks, take control measures, and prevent large-scale spread.
3. Healthcare surge capacity: Preparing for a sudden increase in cases requires capacity building in the healthcare system. This includes identifying and setting up additional facilities, training staff, and ensuring sufficient resources to ensure effective patient management.
4. R&D vaccines and therapeutics: The preparedness–response phase involves significant investment in the research and development of vaccines and therapies specific to the pathogen in question. This effort aims to ensure that once the pathogen is identified, there are therapeutic and preventive options ready to be implemented.
5. Management of long-term impacts: In addition to immediate response, the preparedness–response phase includes consideration of long-term public health impacts. This involves managing the psychological, social, and economic aspects, as well as planning for the long-term recovery of the affected community.

**Figure 3:**
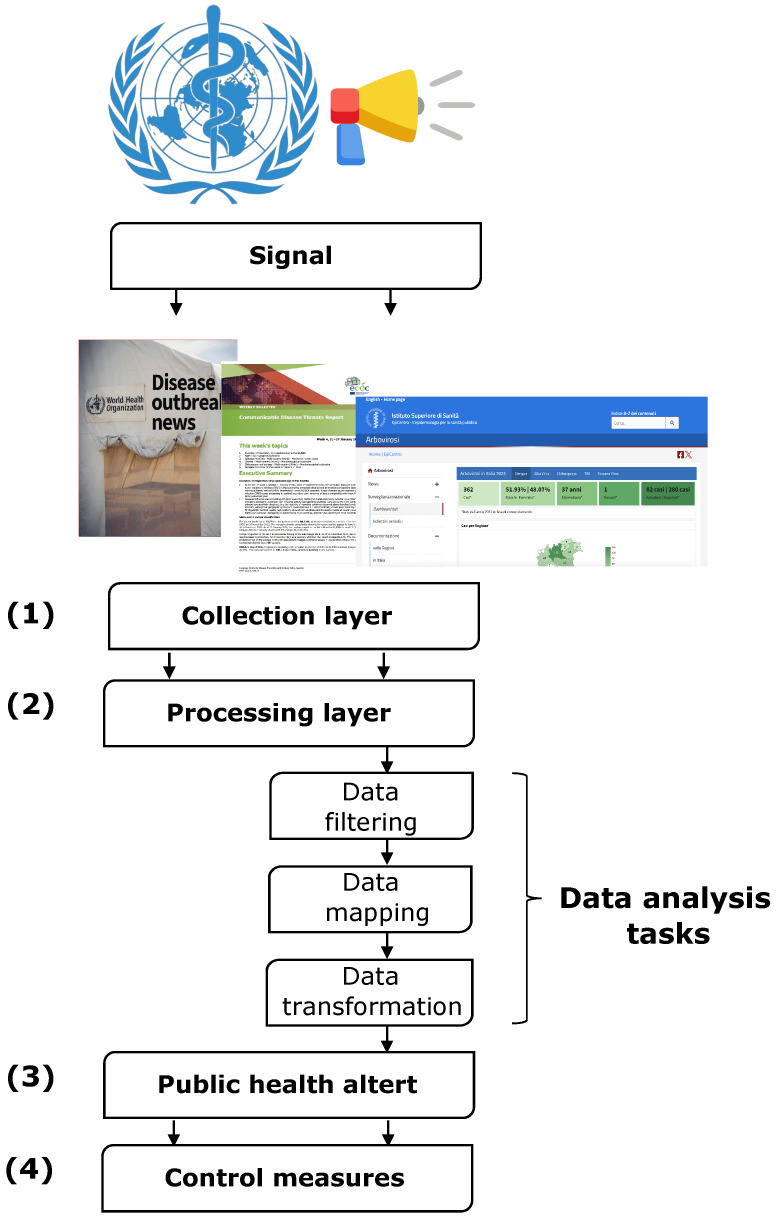
ECIP framework.

The ECIP framework comprises five standard steps, as illustrated in Figure 3. It is designed to be applicable to any situation and can be employed at various levels within the public health system. In the context of a specific situation, such as an outbreak, these steps may be revisited multiple times, forming an iterative process. This iterative approach enables the incorporation of new developments, fostering continuous improvement in the decision-making process. Following an alert of a potential threat to global health from the World Health Organization (WHO), the first step is data collection, which refers to unstructured data collected from intelligence sources of any nature. Collecting a large amount of information from a variety of sources requires ECIP to filter, process, and summarize the information to exclude reporting errors and ensure the robustness of the datasets (step 2). In particular, data mapping is a critical task because it involves designing a data schema and developing a well-defined and standardized structure that outlines the format and relationships within the dataset. The data schema serves as a model, ensuring consistent organization and representation of information. In addition, the creation of a comprehensive data dictionary is critical. This involves creating a detailed reference guide that provides clear definitions and specifications for each variable or field in the dataset. The data dictionary promotes a shared understanding of terms, encouraging uniform interpretation throughout the system.

Once the database has been constructed, the nature of the signal, the extent of the problem, the type(s) of disease potentially involved, and the population affected are checked. For example, interactive maps are made to see how a particular phenomenon has developed in the past, how it is progressing, and whether it is a possible threat in the future, based on mathematical models (step 3).

Finally, ECIP focuses on the practical implementation of the control measures derived from the health alert analysis in the previous phases (step 4), the key actions of which include (i) communication strategies, which is to implement clear and effective communication strategies to disseminate information about the threat, control measures, and preventive actions for the public, health workers, and other stakeholders; (ii) stakeholder coordination, which is to collaborate with various stakeholders, such as government agencies, nongovernmental organizations, and international bodies, to improve the collective response and maximize the impact of control measures; and (iii) monitoring and evaluation, which is to establish an ongoing monitoring and evaluation system to assess the effectiveness of the implemented control measures. This involves continuously analyzing data, adjusting strategies as needed, and ensuring a dynamic response to changing situations.

## 3. Limitations

Currently, ECIP has some limitations that may affect its effectiveness and usefulness:

1. ECIP bases much of its information on reports published by national and international health institutions and organizations. Although this provides a constant flow of epidemiological data, the dependence on external sources may make the platform vulnerable to delays or gaps in reporting. In addition, the accuracy and reliability of the data depend on the quality and timeliness of the reports provided, which may limit ECIP’s ability to provide a complete and up-to-date view of the epidemiological situation.
2. Because of the complexity of the epidemiological data and analyses on ECIP, users must have a solid understanding of the tools and methodologies used to analyze these data. This can be a barrier to access for those without specific training in epidemiology or data analysis, thus limiting the use of the platform to an inner circle of experts.
3. ECIP’s user experience capabilities may not be optimal for all users, especially those who prefer a more graphical and intuitive presentation of epidemiological data. Although the platform provides a number of data repositories that sometimes also contain tools for analyzing and interpreting data, some users may find the interface unintuitive or difficult to navigate. Currently, the platform lacks interactive features and advanced visualization tools that allow users to explore data more dynamically and intuitively.

## 4. Conclusions and Future Work

In the face of persistent challenges posed by pandemics and emerging infectious diseases, the need for robust preparedness and response mechanisms is more evident than ever. In the coming years, ECIP is poised to increase its functionality by enhancing routine activities related to epidemic risk monitoring and assessment. This includes integrating additional data streams to enrich the platform’s capabilities, automating the process of collecting and saving information through the use of APIs, and ad hoc scripts for each database. In addition, by directly involving representatives of national health authorities, ECIP aims to establish a collaborative framework to ensure a continuous exchange of information and expertise, no longer playing a passive, wait-and-see role of publishing bulletins. This proactive approach will seek to foster a synergistic relationship between experts and users, promoting shared responsibility for public health preparedness.

ECIP aspires to be a pioneer in the field of epidemic preparedness, equipping future professionals with the skills and knowledge needed to effectively address emerging health challenges. Recognizing the imperative of developing forward-looking policies, the platform strives to empower professionals in the field. By enabling comprehensive data analysis and fostering collaboration, ECIP seeks to contribute not only by understanding current disease dynamics but also by anticipating future challenges. The platform’s commitment to leading by example goes beyond the technical aspects and includes creating an agile and proactive mindset within the public health community.

A key aspect of ECIP’s vision for the future is to ensure the interoperability of different surveillance systems. ECIP supports the need for a shared language among different systems, emphasizing the importance of common standards. This commitment to interoperability aims to simplify communication and data exchange, crossing organizational and geographic boundaries. By speaking the same language, these surveillance systems can collectively contribute to a more cohesive and effective global response to emerging health threats. To ensure more widespread adoption of the platform and more effective collective participation, a new section is planned in the coming months that will provide interactive graphical tools that allow users to dynamically explore the data, tailoring analyses to their specific needs and interests. This enhancement aims to increase the usability of the platform, ensuring that epidemiological data analysis is accessible even to those without public health experience. This new functionality will significantly increase the value of ECIP as a resource for public health emergency preparedness and management by expanding access to and understanding of critical public health information. In collaboration with the Italian open data portal, testing of this functionality is underway. For example, in the case of West Nile (http://tinyurl.com/west-nile-example, accessed on 28 February 2024), users can click on a file within the dataset to preview the data in tabular format and select the most suitable chart type.

The recent COVID-19 health emergency underscored the interconnected nature of global health. ECIP recognizes the growing sense of cohesion and interconnectedness among diverse communities. The platform envisions a future in which collaborative efforts become the cornerstone of shared strategic goals. By promoting a collective understanding that individual actions impact everyone’s life, ECIP supports a choral effort in which nations, organizations, and professionals come together to address common problems. This collaborative spirit will be essential to achieving resilience and preparedness on a global scale.

## Data Availability

Data that support the findings of this study are available at https://tinyurl.com/episorveglianza (accessed on 25 January 2024).

## References

[1] Dron L., Kalatharan V., Gupta A., Haggstrom J., Zariffa N., Morris A.D., Arora P., Park J. (2022). Data capture and sharing in the COVID-19 pandemic: A cause for concern. Lancet Digit. Health.

[2] Cake C., Ogburn E., Pinches H., Coleman G., Seymour D., Woodard F., Manohar S., Monsur M., Landray M., Dalton G. (2022). Development and evaluation of rapid data-enabled access to routine clinical information to enhance early recruitment to the national clinical platform trial of COVID-19 community treatments. Trials.

[3] Kherabi Y., Launay O., Luong Nguyen L.B. (2022). COVID-19 vaccines against Omicron variant: Real-world data on effectiveness. Viruses.

[4] World Health Organization Disease Outbreak News (DONs). https://www.who.int/emergencies/disease-outbreak-news.

[5] European Centre for Disease Prevention and Control Disease Surveillance Reports. https://www.ecdc.europa.eu/en/search?s=&f%5B0%5D=categories%3A1253.

[6] Istituto Superiore di Sanità Health Topics. https://www.epicentro.iss.it/en/index/infectious-diseases.

[7] Branda F., Abenavoli L., Pierini M., Mazzoli S. (2022). Predicting the Spread of SARS-CoV-2 in Italian Regions: The Calabria Case Study, February 2020–March 2022. Diseases.

[8] Branda F., Mazzoli S., Pierini M., Ciccozzi M. (2023). Trends and Spatiotemporal Patterns of Avian Influenza Outbreaks in Italy: A Data-Driven Approach. Infect. Dis. Rep..

[9] Mingione M., Branda F., Maruotti A., Ciccozzi M., Mazzoli S. (2023). Monitoring the West Nile virus outbreaks in Italy using open access data. Sci. Data.

[10] Branda F., Mazzoli S. (2023). The importance of rapid and robust availability of epidemiological data for real-time mapping of the risk of avian influenza a (H5N1) spread. Pathog. Glob. Health.

[11] Branda F., Pierini M., Mazzoli S. (2023). Monkeypox: Early estimation of basic reproduction number R0 in Europe. J. Med. Virol..

[12] Cunningham A., Markotter W., Mettenleiter T.C., Adisasmito W.B., Almuhairi S., Barton Behravesh C., Bukachi S.A., Casas N., Cediel Becerra N., Charron D.F. (2023). Prevention of zoonotic spillover: From relying on response to reducing the risk at source. Plos Pathog..

